# Turns and Turn-Related Falls: Stepping on a Rotating Platform as a Potential Approach to Foster Adapted Muscular and Neural Plasticity in Fragile Persons and Patients with Motor Impairment

**DOI:** 10.3390/brainsci16070762

**Published:** 2026-07-20

**Authors:** Shashank Ghai, Marco Schieppati

**Affiliations:** 1 Department of Political, Historical, Religious and Cultural Studies, Karlstad University, 65188 Karlstad, Sweden; 2 Centre for Societal Risk Research, Karlstad University, 65188 Karlstad, Sweden; 3 Independent Researcher, 20123 Milan, Italy; marco.schieppati@icloud.com

**Keywords:** ageing, frailty, curved walking, falls, gait rehabilitation, movement disorders

## Abstract

**Highlights:**

**What are the main findings?**

- Falls are exacerbated by a sedentary lifestyle and frailty accompanying the ageing process.
- Falls during changes in direction are more dangerous than those during straight-line walking and carry a higher risk of femoral fractures.
- Curved-path walking is an understudied aspect of locomotion that warrants targeted intervention. We summarise here some characteristics of curved walking and turns and their neural control.

**What are the implications of the main findings?**

- A rotating platform on which people march on the spot whilst holding a stable support may improve strength and neuromuscular coordination by selectively activating the deep pelvic muscles responsible for lower limb rotation.

**Abstract:**

Falls remain the leading cause of injury-related death among older adults, with turning accident often producing the most severe consequences, such as hip fractures and a resulting decline in independence. Despite curved-path walking accounting for the majority of daily locomotion, most interventions insufficiently target the coordinated action of lower limb, pelvic and trunk muscles underlying safe turning. This leaves a large proportion of older adults and patients with motricity problems and movement disorders at heightened risk of life-altering injuries. Addressing this gap requires innovative strategies that proactively build on recent physiological research and improve turning strength and neuromuscular control of steering. Gait problems have often been addressed through the use of linear treadmills, which emphasise the symmetry and automaticity of walking but ignore the fact that more than half of daily locomotion involves deviations from the trajectory. In this perspective article, we review the differences between linear and curved walking. We also put forward the hypothesis that a turntable rotating parallel to the horizontal plane represents a potential solution. Such a device could allow individuals to step in place while the surface moves beneath them, simulating the natural rotation of legs, pelvis, and trunk involved in turning. The exercise is designed to train the muscle synergies and proprioceptive responses critical for turning, while minimising stress on vestibular systems, and enhancing task-specific neuroplasticity and proprioception.

## 1. Ageing and Falls

A plethora of complex weaknesses in the neuromuscular mechanisms contribute to reductions in walking function associated with advancing age [1,2]. Loss of muscle fibres begins around the age of 50, such that about 50% of all muscle fibres are lost by 80 years of age [3,4]. Both common and less common conditions, including sedentariness, gluteal amnesia [5], obesity, blurred vision or poor visual scanning of the environment [6], cognitive decline and attention disorders [7,8], and pain [9], all can add up and increase the risk of falls.

Other conditions include diabetes and peripheral neuropathy [10], alteration of the blood–brain barrier [11], impaired vestibular function [12], hearing loss [13], osteoporosis [14], and cardiovascular problems [15]. Malnutrition [16] and hyponatremia [17] are often underdiagnosed, as is anaemia, which affects more than 10% of the older population [18]. Polypharmacy, including chemotherapy [19], further contributes to balance and gait problems [20].

Even in high-performing older adults who retain subjective confidence in their balance [21], locomotor control becomes less automatic [22]. They may also suffer from a deficit in the central integration of varying sensory information for reconfiguring the postural set [23], so that self-assessed perceptions of balance may not reflect actual capabilities [24,25] and may lead to falls. Furthermore, older adults subjected to postural disturbances exhibit a longer initial phase of destabilisation and take longer to regain coordinated stability in the centre of mass over the support base [26].

Particularly worrying is the cyclical nature of falls. Among patients hospitalised for a fall, a large proportion return to the hospital for a subsequent fall within 6 months [27]. Hip fracture is both a consequence and a cause of sarcopenia, and not all femoral fractures are equal, particularly in the light of the rehabilitation costs [28]. As demographics shift towards an ageing population, the burden of care for patients with hip fracture is expected to grow [29]. Importantly, up to 76% of these patients experience functional loss despite receiving standard rehabilitation as part of their fracture management [30].

## 2. A Complex Task: Walking and Turning

Non-linear paths are risky for older adults and frail individuals, yet these trajectories account for more than half of total daily locomotion [31,32,33]. Older adults walk with reduced velocity and may adopt a cautious attitude when steering, partly as a consequence of fear of falling [34]. Falls occur predominantly during changes in walking trajectory and slips [35], and those during turns are the most dangerous, as they are more likely to result in femoral and hip fractures than falls during straight-line walking [36].

Locomotion along curved trajectories requires fine coordination among body segments [37]. This reflects the asymmetric motion of the two antimeres, whereby the step length is shorter on the inside of the curved trajectory [38]. The trunk leans inward to counteract the centrifugal force. Since we do not push against a wall while walking in anticipation of the turns, and no external force like a gush of wind or a nudge from a friend moves us in the desired direction, the foot sole contact point is the only point of contact through which the ground reaction force can affect the path’s trajectory. That point is, in fact, appropriately slightly shifted compared to linear walking in order to create and exploit gravity and produce the centripetal force and control the inward fall of the body, as well [39,40]. Turning is accompanied by dedicated mediolateral anticipatory adjustments. Of note, these are impaired in Parkinson’s disease [41].

Accordingly, brain activation for imagined straight walking and imagined walking along a curved path has been investigated [42,43]. In curved walking, a shift in activation of the basal ganglia and enhanced activity in certain cortical areas have been observed, alongside deactivation in others. Of note, considering that turning and walking are tasks where deliberation and motor planning are intertwined [44], a sort of dual-task condition occurs that is associated with an increased risk of falls [45,46]. Brain stem centres specifically involved in the coupled control of posture and progression may lose their automatic pairing during turning in pathological states [47].

## 3. Muscles Involved in Turning

The pelvic muscles assisting the intra- and extra-rotation of the lower limbs have been depicted by Neumann and Kelly [48] and their function discussed by Dostal, et al. [49]. These are the short external (lateral) thigh rotators: gluteus maximus, superior and inferior gemelli, quadratus femoris, obturator internus and externus, and piriformis. The internal (medial) rotators of the femur at the hip are the tensor fasciae latae, anterior-most fibres of gluteus, adductor longus and adductor brevis. Several other muscles in the lower limb are active to a different extent during turns than straight walking [50]. However, their contribution to turns of the leg and thigh is likely to be ancillary or just incremental [51]. By analysing the data from a simulation study of a fair number of muscles in different body parts, including the gluteus, Ventura, et al. [52] confirmed the occurrence of clear-cut task-specific synergies for turning. Simulation studies also showed that the muscles that stabilise the hip and ankle behave differently during curved walking with respect to straight walking [53], though deeper muscles were not included in the simulation.

Admittedly, studying the intra- and extra-rotator muscles deep in the pelvis by recording the muscle electrical activity through electromyography (EMG) is not easy, although it is crucial, for instance, prior to and after hip replacement surgery [54,55]. Their contribution goes unaddressed even in a recent study of repeated turns in competitive skiing, despite their presumed relevance [56], and in a pioneering EMG study, where Hase and Stein [57] pointed to the role of the gluteus in rapidly changing walking direction. The force production of the deeper muscles can be measured via simple dynamometry, though [58,59]. Interestingly, computed tomography and dynamometry of thigh and hip muscles distinguished fallers from non-fallers, with gluteal muscles proving to be most affected, even though deeper pelvic muscles were not included, yet again [60].

The trunk muscles play a major role in facilitating and coordinating movements during walking [61], functioning as both mechanical stabilisers and sensory integrators. This action is essential for balance during changes in direction. Multifidus, transversus abdominis, and internal and external obliques form a functional unit that responds with increased activity during isometric hip rotation [62]. The erectores spinae play a role in halting forward momentum and initiating axial rotation during turning. Accordingly, poor control of trunk kinematics in older adults results in increased fall risk [63].

Beyond mechanical stabilisation, trunk muscles provide sensory input that actively assists in steering locomotion. Schmid, et al. [64] showed that proprioceptive feedback from trunk muscles contributes to the control of walking trajectories during turns. As a matter of fact, unilateral proprioceptive input by vibration from all the axial muscles has prompt effects on stance and locomotion direction, producing turning [65]. This is in keeping with the notion that axial muscles’ proprioception, from pelvis to neck, has crucial influences on body posture, orientation, perception of motion, and stepping characteristics [66]. It is, therefore, reasonable to propose that the neuromuscular adaptations induced by stepping on the rotating platform extend beyond turning *per se* and may contribute to postural control more broadly. The repeated engagement of lower limb rotator muscles and the continuous proprioceptive inflow from axial muscles during platform stepping are precisely the inputs that govern upright stability during both stance and locomotion. Additionally, the relevance of trunk proprioception is also true in patients with Parkinson’s disease (PwPD) [67], who are able to correctly integrate and exploit a rhythmic vibration-induced proprioceptive inflow to produce entrained oscillations in the centre of mass while standing and walking, with gait velocity also enhanced [68,69].

## 4. Interventions

There is a plethora of suggestions for helping older adults avoid falls (e.g., [70]). Exercise interventions do produce significant benefit across multiple fall-related outcomes [71]. Tai Chi can be helpful for improving balance in older adults at risk of falling, although its benefit in complex walking conditions remains untested [72]. Dance can be effective in preventing falls, but it is unlikely to reach most patients in need [73].

While various interventions show promise [74,75,76], the best-practice principles underlying successful rehabilitation dictate that exercises and interventions be task-specific and closely match the individual’s specific injury or condition, as well as the functional activities they need to perform [77]. This requires targeting specific muscles, movement patterns, and functional tasks to promote optimal recovery and return to desired activities. Furthermore, any treatment must be safe [78], easy, non-invasive, effective, scalable, and grounded in basic physiological principles.

A frequently used gait training device is the linear treadmill. An evolution of it is the so-called split-belt treadmill, in which two bands move at different speeds, producing major adaptations and complex cortico–muscular changes [79], but gait is always in a straight line. Despite building strength, these modalities may not translate to real-life turning tasks and fail to incorporate the element of coordinated activity of the muscles in the lower limbs, pelvis, and trunk, along with the neural coordination involved in turning. Beyond rehabilitation, the same gap is evident in sport training. Athletes in field sports routinely execute rapid directional changes that depend on the same intra- and extra-rotator muscle groups and neuromuscular synergies described above [80]. Conventional strength and conditioning programs largely prioritise linear movement patterns, leaving these rotator muscles undertrained and potentially predisposing even fit individuals to injury during turns [81]. There are also complex, wearable, motorised exoskeletons that can aid activities of daily living, but the production of curved gait is not normally envisaged [82,83,84]. Furthermore, they are not widely adopted in clinical settings due to the ‘disconnect’ between the needs of exoskeleton users and the positions of the engineers designing the devices [85], and entail a number of risks that are not always evaluated [86]. Omnidirectional treadmills help adaptation to a moving support surface and to postural perturbations, but the generalisability of the findings to the real world for fall prevention has not been proven [87].

Based on these considerations, stepping on the spot while spinning around may represent the simplest possible task that can be easily performed. This approach might appear to be worth testing for its potential to improve turning ability, help prevent falls, and serve as a rehabilitation strategy, without the ‘voluntary’ progression effort of treadmill walking or the need to produce a full stride, which may not always be comfortable for older adults. Certainly, it specifically engages the intra- and extra-rotator muscles of the lower limbs at the pelvis consistently with their function during curved walking, while also stabilising the trunk.

## 5. Could a Rotating Platform Help?

**Figure 1:**
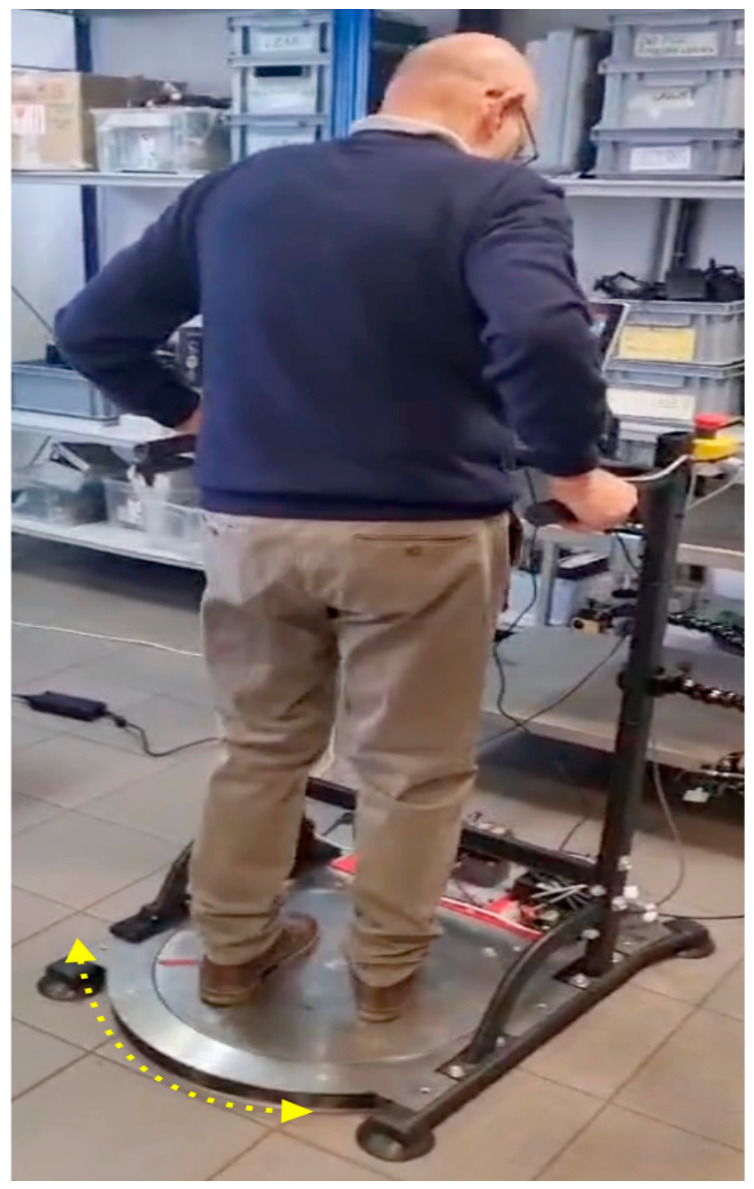
The conceptual design of the rotating platform. The user steps in place on a horizontal disc while holding a fixed handrail. The disc rotation induces intra- and extra-rotation of the lower limbs in sequence, with the trunk and head stabilised in space. The yellow curved arrow illustrates the platform’s rotational capabilities in both clockwise and counterclockwise directions.

Stepping on the spot and spinning along the vertical axis of the body favours dynamic control of posture and coordination between the legs and trunk [88]. The big problem is the vestibular stimulation and vertigo linked to the head rotation. It is, therefore, necessary to change the reference system: stepping in place on a motorised rotating platform, while maintaining a steady body and head orientation in space, avoids head rotation whilst producing the desired leg longitudinal rotations. A device has been designed (Figure 1) to selectively promote the intra- and extra-rotation of the lower limbs, exercising the muscles that provide the activity necessary for the generation of curved walking.

The rotation along the major axis of the lower limbs during the stationary stepping in place is produced by the platform, similar to a turntable, and occurs through two mechanisms: passive rotation during foot stance and active rotation during the foot/leg lifting phase. The same movements occur for both legs and feet, but they are out of sync by half a beat and not symmetric because one foot/leg is extra-rotated when the other is intra-rotated. Importantly, users need not learn unusual or unfamiliar movement patterns because the instruction is simply to step in place. Stepping on the rotating platform is expected to exert minimal additional load on the hip [89], unlike treadmill walking, and little impact on the heel because it lacks the ‘forward thrust’ and the heel strike imposed by walking on a treadmill. The rotating platform does not require displacement of the body and feet relative to the translating surface, eliminating the active braking of body weight at heel strike and prior to the so-called push-off [90].

A limitation of such a rotating platform is the missing inward trunk inclination that occurs during normal curved walking [39] and is proportional to the body angular velocity. This is because the body lies practically along the vertical through the turntable pivot and has no angular velocity, so that the centrifugal force is negligible, and no centripetal force needs to be produced. Importantly, the small 50 cm diameter of the turntable, with the feet very close to the centre of rotation, elicits the greatest possible angular rotation of the feet and lower limbs, compared to walking along the circumference of a larger platform.

Notably, the feet rotation angle on the horizontal plane depends both on the angular velocity of the platform (from 0 to 60°/s) and on the stepping frequency. A slow stepping rate at a fast angular velocity implies a long-duration stance phase, during which the foot is passively translated/rotated to a large extent. Conversely, a high stepping rate minimizes the duration of the transport period of the stance foot, therefore the lower limb rotation, because the stance phase is short and is rapidly interrupted by the next foot lift. By operating an acoustic metronome built into the device, an approach commonly used in PwPD [91,92], and by modulating the platform angular rotation, the platform motion can, therefore, affect the pattern of the lower limb rotation and feet placement.

## 6. Operating Description

Such a device offers significant versatility in its programming parameters. The angular velocity of the rotating disc can vary to adapt the rotation to the subject’s abilities and treatment stage, from slow to incrementally faster speeds, either within a session or across repeated sessions. The rotation may have constant or variable angular velocity. It can be continuous clockwise or counterclockwise, or have periods with increasing and decreasing angular velocities, with reversal of the direction of rotation. The duration of the rotation period can be short or prolonged, configuring a ‘gait endurance’ type of training. Short, rapid, impulsive rotations of the platform could be administered as well. In different contexts, perturbations have so far only been described during stance [93,94,95], forward-inclined posture [96], linear treadmill walking [97,98], and curved path walking via slippery shoes [99]. However, none of these specifically perturbs the lower limb rotator muscles through sudden rotation of the stance limb.

**Figure 2:**
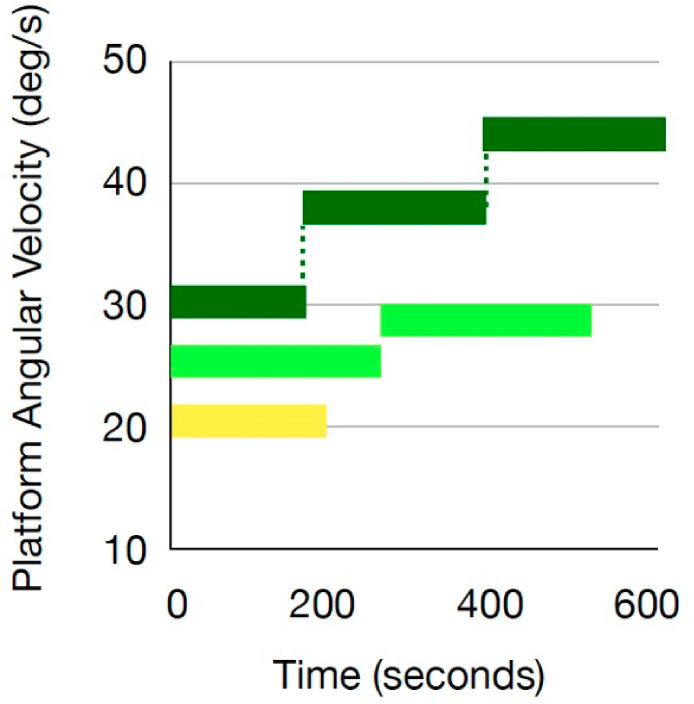
An example of the administration of the training/treatment. The bars represent platform rotation velocities (ordinate) and durations (abscissa) on the 1st (yellow), 5th (light green), and 10th (dark green) training days. For simplicity, only clockwise rotations are illustrated, though both clockwise and counterclockwise rotations would be used in the same session. The figure shows progression in both rotation velocity and duration within a session (same colour) and across training days. The dashed vertical lines indicate the transition between successive rotation velocities within the same training session. A familiarisation trial (yellow) may be performed at a very slow velocity (even much slower than 20°/s) and a short duration according to patient tolerance. A maximum duration of 10 min would be reached in later sessions (dark green).

Figure 2 illustrates an example of treatment administration over time and of intensity progression across subsequent sessions, showing both the velocity and duration of the platform motion, within a session and across days. Familiarisation may require some time at the beginning of the treatment, which would start with the low angular velocity of the turntable. This time period is expected to be briefer than with linear treadmills, where in older adults, treadmill walking may not reach a velocity nearing that of overground walking even after several minutes [100]. Despite the fixed handrail and the low centrifugal demand afforded by the small turntable diameter, imbalance remains a relevant concern, particularly during early sessions, at higher angular velocities, or during the brief, deliberately delivered angular postural perturbations, and especially in frail older adults or patients with impaired postural control. It is known that tripping risk increases in older adults following 6 min of fast walking on a linear treadmill [101]. Close supervision, gradual familiarisation at low velocity, and a harness should, therefore, be considered standard practice, at least until an individual’s tolerance and stability on the device have been established.

Similarly, fatigability is an important consideration, particularly in older adults. During the initial treatment sessions, fatigue of the pelvic muscles involved in stepping on the platform may develop after a few minutes of exercise. Because this sensation may be unfamiliar, it could transiently influence locomotion immediately after treatment and should, therefore, be appropriately monitored. To minimise potential adverse effects, participants should remain standing quietly on the stationary platform for a brief period before resuming normal activities. Fatigue of the axial muscles can also disrupt spatial orientation and impair the perception of slow movements, both in the presence and absence of vestibular stimulation [102,103], potentially increasing the risk of instability during tasks requiring accurate spatial perception. Consequently, the potential effects of muscle fatigue following rotating-platform training warrant investigation, as this intervention may introduce fall-related risks that differ from those associated with conventional treadmill-based rehabilitation [104], and optimal training parameters should be informed by dose–response data that are not yet available for this device.

In sum, the rotating platform concept is potentially adaptable to individuals’ needs, with sessions tailorable to personal factors including age, frailty, compliance, fatiguability, interaction with the caregiver, clinical condition, severity of gait impairment, etc.

Equipping the device with video cameras could capture the lower limbs and feet motion during stepping, with the live images displayed on a tablet placed in front of the user. This real-time visual feedback may enhance awareness of foot placement and rotation, reinforcing sensorimotor learning [105,106]. This feature may be particularly relevant since visual cueing strategies can improve turning performance and gait control in PwPD, likely by increasing attentional engagement and compensating for impaired automatic motor control [107].

For both research and clinical practice, the device can be equipped with supports of different thicknesses and consistencies applied to the turntable, encouraging lifting of the legs and preventing the feet from dragging [108]. Wearable units could be implemented as well, both for monitoring kinematics of the turning body [109] and for measuring physiological parameters of the exercising subjects [110].

## 7. Is Neuromuscular Coordination Boosted?

Stepping in place is not a trivial motor act. It engages spinal central pattern generators that are highly sensitive to proprioceptive inflow and constitute a privileged substrate for inducing locomotor plasticity at both spinal and supraspinal levels [111,112]. The alternating loading and unloading of each limb during stepping maximises the contrast between intra- and extra-rotatory proprioceptive signals, potentially rendering the input from the rotating platform richer and more behaviourally relevant than passive limb rotation or static standing could produce. Turning while stepping in place stimulates neuromuscular coordination and neural plasticity [113], alongside bolstering muscular strength. The underlying neural mechanisms involve sensory feedback from intramuscular receptors that plays a vital role in learning and reinforcing specific movements (see Marasco and de Nooij [114], for a recent review).

The capacity for incorporating proprioception manifests itself typically in a phenomenon of persistent involuntary turning in place with eyes closed, which builds up immediately at the end of the platform rotation and persists for several seconds, highlighting the retention of a newly acquired coordination [94,95]. This effect emerges after a few minutes of training, probably because the large degree of the lower limb rotation angle elicits a large and distinct proprioceptive input. In the past, a large rotating drum had been described, where subjects walked on the periphery of it with their eyes open. Thereafter, blindfolded subjects involuntarily walked around a circular path without being aware of their curved trajectory, a phenomenon termed podokinetic after-rotation (PKAR) [115,116]. The same phenomenon has been found after prolonged stepping-in-place on the centre of a large rotating turntable [117]. The post-effect elicited by stepping in place on the rotating platform has a non-negligible duration and is similar in time and amplitude to the germane phenomenon elicited by simply stepping in place on stable ground and turning around voluntarily for a while. Both continuous voluntary turning on the spot [94] and continuous stepping on the rotating platform exert similar PKAR effects. Remarkably, the ‘memory’ of the motion persists for several seconds without eliciting dizziness, suggesting possible deep central neural integration at the spinal level, reminiscent of the Kohnstamm phenomenon [118,119]. This is consistent with reports that direction-dependent post-effects on quiet stance occur after linear treadmill locomotion [120,121], and effects on body posture and/or stepping are observed after prolonged muscle contractions, as well [102,122]. The neural origin of this phenomenon is further corroborated by the finding that the PKAR is transiently enhanced or reversed (depending on the stimulated body side) by unilateral axial trunk muscle proprioceptive stimulation by vibration [123]. Clearly, this effect cannot simply depend on the increase in strength of the muscles responsible for turning due to the training. Rather, it implies a storage and recollection of the nervous activity controlling the coordination of movement, suggesting that a learned pattern survives in the nervous system after prolonged turning and podokinetic stimulation (both voluntary and platform-induced). Hence, such exercise emphasises the plasticity of the sensorimotor system and may elicit spinal and supra spinal adaptations [112,124].

Walking on rotating platforms constitutes an intriguing form of sensory conflict: the sense of sight and the vestibular system are stimulated little during the platform rotation while holding onto a stable frame, in a way not much different from during linear walking, while the proprioceptive inflow definitely informs the nervous system of the turning mode. The interaction of visual, vestibular, and proprioceptive inputs has been the object of pioneering investigations [125,126]. Proprioceptive–vestibular interactions, coupled with corollary discharge of a motor plan, certainly occur when stepping on the rotating platform. This would allow the brain to distinguish actively generated from passive head movements [127]. However, Earhart, et al. [128] showed that complete absence of vestibular input has only an initial, albeit clear-cut, effect on the PKAR, speaking for a major role of the proprioceptive input. Admittedly, understanding the role of sensory mismatch while stepping on the rotating platform requires further investigation, even if the various tasks in which the Kohnstamm phenomenon has been shown to favour the proprioceptive input. Ivanenko, et al. [129] showed that, when subjects opposed a rotational force applied to the pelvis for about 30 s, they walked along a curved trajectory in the direction of the preceding torsion. Understanding the role of the sensory mismatch while stepping on the rotating platform and in the post-effect requires further investigation.

## 8. A Potential Intervention: Parkinson’s Disease

Treatment by podokinetic stimulation can be exploited in clinical populations to improve locomotor turning. As mentioned, gait, balance and turns impairments are typical of PwPD [130] as an indication of decreased gait complexity [131]. Walking along curved trajectories highlights impaired gait control even in on-phase patients [132]. The fine control of curved walking, when one cannot rely on the automatic mechanisms of straight-line walking [133,134,135], could also be affected by their rigidity [136] and their slowness in voluntary muscle relaxation [137].

Freezing of gait (FoG) in PwPD, with the associated risk of falling, is a common event in the advanced disease and is exacerbated by turns [138,139]. Advanced neuromodulation techniques have shown some success in reducing freezing. Neuromodulation by non-invasive magnetic cortical stimulation is an example thereof. However, once subjected to criticism, neuromodulation seems to have produced largely ambiguous effects [140,141]. Spinal cord stimulation through a lead implanted in the epidural space has been proposed as well. A recent trial with stimulation at the thoracic level found no evidence of a clinically relevant improvement in gait and FoG [142]. Returning to more fundamental mechanisms, given the relevance of impaired balance control during turns and steering of gait in PwPD, the abnormal modulation in amplitude and timing of postural reactions and anticipatory adjustments [143,144] should be studied further to assess whether these deficits can have an effect on turning and be improved with appropriate training.

Recent review articles addressed the efficacy of physical interventions designed to reduce falls in PwPD [135,145,146]. All-type exercise interventions had minor or moderate effects on adverse events, and it was not clear whether exercise was a cost-effective intervention for fall prevention. Additionally, it has been shown that robotic (linear) gait training is not superior to conventional treadmill training in PwPD [147]. But exercise remains a pivotal element for training [148,149], and PwPD retain a sufficient capacity for motor learning [150,151,152], even if proprioception is not optimal [153]. Interestingly, a pilot investigation found promising results on recovery of locomotion speed by using a rotating platform in PwPD: post-training, the velocity of walking increased, more so for circular than linear trajectories [154]. Moreover, preliminary data have shown that the platform–rotation treatment seems to reduce freezing, as well [155].

## 9. A Brief Look at Other Conditions That Would Possibly Benefit from the Rotating Platform Treatment

Many disorders of the nervous system involve gait disturbances, including difficulties in following a curved path while walking [156]. It has been known for many years that the ability to navigate curves is impaired in mild cognitive impairment [157,158]. This may occur either as part of a generalised movement disorder or as a specific feature associated with the lesion, the underlying condition, or both [159].

Paresis and spasticity after stroke limit walking mobility, both during linear progression and during turning and changes in direction [160]. Curved locomotion is defective in patients who have had a stroke [161,162], and curved-path gait training results in greater improvement in gait ability than general gait training [163,164].

Children with cerebral palsy also show abnormalities in trunk–pelvis relation during walking. Their turning gait is different compared to that of typically developed children, and internal rotation gait is common [165]. Hence, gait-pattern-specific biomechanical shortcomings are present, including impaired coordination between lower limb segments and trunk mobility [166], which produce turning problems in addition to linear-walking impairments [167].

Incomplete spinal cord lesions may spare basic locomotion [168], but most patients are at risk of falling because of changes in sensation and motor function, which differ from person to person, and require personalised interventions [169,170]. A proposal to include curved walking exercises in the rehabilitation program for patients with SCI was put forward a long time ago [171], but the suggestion has not been followed.

Multiple sclerosis shows a huge variety of clinical manifestations, but turning is often affected due to short strides more than changes in cadence, possibly due to problems in cortical circuits [172]. Studies on the potential improvement of walking and turning by targeting dynamic balance in MS are wanted [173,174].

We have previously mentioned hip fractures as a serious consequence of falls, particularly in frail persons [175]. Interestingly, pre-habilitation has been proposed to enhance recovery after total hip arthroplasty, by means of which tolerance to surgical stress and supporting postoperative recovery is expected to improve [176]. A recent systematic review has addressed this issue and found moderate evidence that several exercises can be recommended [177]. The effect of the innovative training proposed here was understandably not included.

## 10. Conclusions

Falls are common, costly, and preventable, bearing extremely serious complications. Effective prevention requires more than just basic, regular exercise. When individuals have difficulty with turning movements and are at risk of falling, whether due to ageing, injury, or neurological conditions, exercises that specifically target the lower limb rotation mechanisms may be beneficial. Engaging in this activity can be easily tolerated and may not represent a barrier even to older or frail people with fears of falls.

As a Perspective article, this manuscript puts forward a conceptual proposal grounded in physiological principles and in findings from related paradigms, such as curved-walking research and rotating-treadmill studies, rather than original data collected using the device described here. Empirical evaluation of this proposal, including EMG, kinematic, safety, training adherence, and fall-risk outcome data, is the necessary next step and is identified here as a direction for future dedicated research.

A device featuring a rotating support onto which subjects step could potentially be implemented into clinical/research practice to improve locomotor turning. This represents an effective, specific approach to training the muscles responsible for changing direction or walking a winding path. If further translational and clinical research supports these preliminary considerations, this approach could potentially contribute to new knowledge on an inadequately investigated motor task and may help support the autonomy and self-sufficiency of many old and frail subjects, as well as patients with motor disabilities.

## Data Availability

No new data were created or analysed in this study. Data sharing is not applicable to this article.
